# A Heparan Sulfate Mimetic RAFT Copolymer Inhibits SARS‐CoV‐2 Infection and Ameliorates Viral‐Induced Inflammation

**DOI:** 10.1002/advs.202411737

**Published:** 2024-12-16

**Authors:** Jiaxin Ling, Åke Lundkvist, Marco Guerrini, Vito Ferro, Jin‐Ping Li, Jinlin Li

**Affiliations:** ^1^ Department of Medical Biochemistry and Microbiology The Biomedical Center Uppsala University Uppsala 75123 Sweden; ^2^ Zoonosis Science Center Uppsala University Uppsala 75123 Sweden; ^3^ Istituto Di Ricerche Chimiche e Biochimiche G. Ronzoni Milan 20133 Italy; ^4^ School of Chemistry and Molecular Biosciences The University of Queensland Brisbane QLD 4072 Australia; ^5^ Australian Infectious Diseases Research Centre The University of Queensland Brisbane QLD 4072 Australia; ^6^ SciLifeLab Uppsala Uppsala University Uppsala 75123 Sweden

**Keywords:** coronavirus, heparan sulfate mimetics, NLRP3, poly(SS‐co‐AA), SARS‐CoV‐2

## Abstract

The high transmissibility and mutation ability of coronaviruses enable them to easily escape existing immune protection and also pose a challenge to existing antiviral drugs. Moreover, drugs only targeting viruses cannot always attenuate the “cytokine storm”. Herein, a synthetic heparan sulfate (HS) mimetic, HMSA‐06 is reported, that exhibited antiviral activities against both the SARS‐CoV‐2 prototype and Omicron strains by targeting viral entry and replication. Of particular note, HMSA‐06 demonstrated more potent anti‐SARS‐CoV‐2 effects than PG545 and Roneparstat. SARS‐CoV‐2 is reported to hijack autophagy to facilitate its replication, therefore boosting autophagy can attenuate SARS‐CoV‐2 infection. It is revealed that HMSA‐06, but not a similar HS mimetic that failed to inhibit SARS‐CoV‐2, can upregulate cellular autophagy flux. In addition, HMSA‐06 was found to robustly block the NLRP3‐mediated inflammatory reaction in SARS‐CoV‐2 infected THP‐1 derived macrophages as evidenced by a reduction in inflammasome formation and the subsequent decreased secretion of mature caspase‐1 and IL‐1β. The HMSA‐06's inflammation inhibitory function is further confirmed using a LPS/ATP‐stimulated THP‐1 macrophage model. Altogether, this study has identified a promising HS mimetic to combat SARS‐CoV‐2‐associated diseases by inhibiting viral infection and attenuating viral‐induced inflammatory reaction, providing insights into the development of novel anti‐coronavirus drugs in the future.

## Graphical abstract

This study identifies a unique HS mimetic, HMSA‐06, that inhibits different types of SARS‐CoV‐2 cell entry and viral replication by boosting autophagic flux, potentially serving as the first line of defense against COVID‐19. Additionally, HMSA‐06 significantly attenuates NLRP3 inflammasome activation, which may help mitigate hyperinflammation during the late stage of SARS‐CoV‐2 infection.

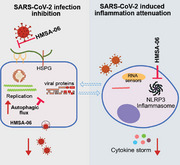

## 1 Introduction

The Coronavirus disease 2019 (COVID‐19) pandemic caused by the novel coronavirus, severe acute respiratory syndrome related coronavirus 2 (SARS‐CoV‐2), resulted in more than 7 million deaths and severely disturbed our socio‐economic activities.^[^ ^1^ ^]^ Despite WHO declaring an end to COVID‐19 as a global health emergency on May 5, 2023,^[^ ^2^ ^]^ SARS‐CoV‐2 is still circulating and the emerging new variants pose a potential threat.

Although the outbreak of SARS‐CoV‐2 could not be predicted, it was not entirely unexpected, as two other epidemics of coronavirus‐associated human diseases, Severe acute respiratory syndrome coronavirus (SARS‐CoV) and Middle East respiratory syndrome coronavirus (MERS‐CoV), had been documented over the past two decades.^[^ ^3^ ^]^ Like SARS‐CoV and MERS‐CoV, accumulating evidence suggests that SARS‐CoV‐2 also has an animal origin, with bats likely being the original host.^[^ ^4^ ^]^ Host diversity, respiratory transmission route, and overlapping habitats with humans dramatically increase the possibilities of new coronavirus variants emerging and spreading across species. Coronaviruses are on the list of pathogens that could spark the next pandemic.^[^ ^5^ ^]^

In response to coronavirus infection, vaccination is still one of the most powerful tools, as demonstrated by the tremendously successful use of mRNA vaccines in mitigating the COVID‐19 pandemic.^[^ ^6^ ^]^ However, the continuous emergence of new variants may cause the efficacy of the vaccine to wane, exemplified by the fact that the Omicron strains break the immunity protection line built by the SARS‐CoV‐2 prototype‐based mRNA vaccines.^[^ ^7^ ^]^ Thus, the use of antiviral drugs for treating COVID‐19 is indispensable regardless of the availability of vaccines. SARS‐CoV‐2 is a large RNA virus encoding 4 structural and 25 non‐structural proteins. Among the structural proteins, the spike (S) protein is responsible for interacting with the cellular receptor(s) and mediating viral cell entry.^[^ ^8^ ^]^ The frequent mutations on the receptor binding domain (RBD) of the S protein led to the emergence of immune‐evading mutants.^[^ ^9^ ^]^ The non‐structural proteins are mainly responsible for SARS‐CoV‐2 replication and create a friendly environment for viral propagation.^[^ ^10^ ^]^ Papain‐like protease (PL‐pro) and a main protease (3CL‐pro) are the two viral proteases encoded by SARS‐CoV‐2 for the cleavage of viral polyprotein to form 16 non‐structural proteins. Therefore, these two viral proteases were utilized as targets for the development of anti‐SARS‐CoV‐2 drugs, especially for the 3CL‐pro which plays a dominant role in viral polyprotein cleavage.^[^ ^11^ ^]^ One of the notable examples is Paxlovid whose antiviral component, nirmatrelvir, is a 3CL‐pro inhibitor.^[^ ^12^ ^]^ Paxlovid has been shown to have potent clinical efficacy in the treatment of COVID‐19 patients by reducing hospitalization and mortality. However, like other antiviral drugs, nirmatrelvir‐resistant viral strains will inevitably appear after large‐scale usage in COVID‐19 patients. Indeed, in vitro experiment has shown that SARS‐CoV‐2 can develop resistance to nirmatrelvir through multiple pathways^[^ ^13^ ^]^ and the resistance has also been observed among naturally occurring mutations.^[^ ^14^ ^]^ Therefore, the development of novel antivirals is essential for the treatment of coronavirus infection diseases and the preparedness for potential future outbreaks.

Paxlovid is recommended for early administration to patients in high‐risk groups after SARS‐CoV‐2 infection, partially because inflammation, rather than the virus itself, is the main factor driving the disease progression in the late stages of infection.^[^ ^15^ ^]^ Inflammasome activation in the infected macrophages is a key driver of COVID‐19 pathology.^[^ ^16^ ^]^ Cytokine storm is closely related to lung damage in severe COVID‐19 patients.^[^ ^17^ ^]^ Activation of the NLRP3 inflammasome was found in the PBMC and the infiltrated leukocytes in lung tissue from patients who died from COVID‐19.^[^ ^18^ ^]^ The severity of COVID‐19 in patients is coupled with pulmonary fibrosis, a condition induced by overreactive NLRP3 inflammasome.^[^ ^19^ ^]^ Several in vitro studies reported that SARS‐CoV‐2 encoded proteins, including the nucleocapsid protein, ORF3a, and NSP6, promote the NLRP3 inflammasome activation.^[^ ^20^ ^]^ Attenuation of NLRP3‐mediated inflammation will alleviate COVID‐19 like pathology by waning the hyperinflammatory reaction.^[^ ^16^, ^21^ ^]^ All those studies suggest the NLRP3‐mediated inflammatory reaction plays a vital role in COVID‐19.

Inhibition of SARS‐CoV‐2 infection coupled with attenuation of the induced inflammation will be an effective treatment for COVID‐19 disease. Actually, Niclosamide, an immunomodulatory drug, has been found to ameliorate the NLRP3‐mediated inflammatory reaction in SARS‐CoV‐2 infected primary human monocytes and suppress SARS‐CoV‐2 replication in hACE2‐Vero cells.^[^ ^22^ ^]^ Roneparstat, an HS mimetic initially developed for cancer treatment, has been reported to inhibit SARS‐CoV‐2 cell entry and significantly reduce SARS‐CoV‐2 S1 protein‐induced cytokine release from human macrophages.^[^ ^23^ ^]^

In the current study, based on the concept of targeting both the virus and the virus‐induced inflammatory reactions, we have identified a promising HS mimetic with the ability to inhibit the replication of different SARS‐CoV‐2 variants and to ameliorate virus‐induced inflammatory responses.

## 2 Results

### 2.1 HMSA‐06 Inhibits the SARS‐CoV‐2 Prototype Infection

**Figure 1:**
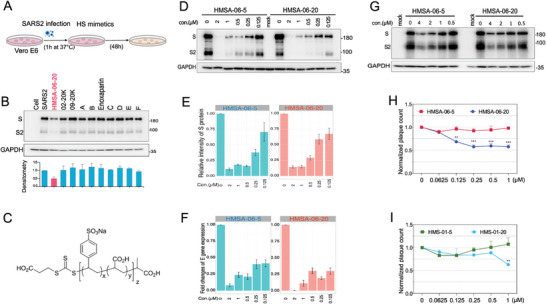
HMSA‐06 inhibits the SARS‐CoV‐2 prototype infection. A) The illustration of screening the effective compounds with capacities to inhibit the SARS‐CoV‐2 prototype replication by post‐viral infection treatment. Vero E6 cells were grown to 70%–80% confluence and were infected by SARS‐CoV‐2 (MOI = 0.01). After 1 h, cells were washed with culture media and fresh media with different HS mimetics were added. Cells were harvested at 48 h post infection for analyzing the SARS‐CoV‐2 infection. B) The representative blots show the expression of SARS‐CoV‐2 spike (S) protein in the presence of different heparan sulfate mimetics (upper panel) and the intensity of S protein (lower panel). The results were shown as mean ± SD from two independent experiments. C) The structure of HMSA‐06. D) The western blots illustrate the expression of S protein in Vero E6 cells in the presence of distinct concentrations of HMSA‐06‐5 or HMSA‐06‐20. E) The intensity of S protein from two independent experiments from (D) was quantified using the ImageJ. F) The mRNA expression of the SARS‐CoV‐2 envelope E) gene was quantified by q‐PCR from the samples in (D). G) The representative western blots of three independent experiments represent the expression of S protein in the presence of different concentrations of HMSA‐06‐5 or HMSA‐06‐20 in Calu3 cells. The number of plaques caused by SARS‐CoV‐2 in the presence of various concentrations of HMSA‐06 (H) or HMS‐01 (I) was assessed by the plaque assay. The data were normalized by the plaque number in the absence of HMSA‐06 or HMS‐01 from two independent experiments (*n* = 2). Statistical analysis was performed using one‐way analysis of variance (ANOVA). ^**^ *p* ≤ 0.01, ^***^ *p* ≤ 0.001.

To screen for effective HS mimetics against SARS‐CoV‐2 infection, we first used Vero E6 cells infected by the SARS‐CoV‐2 prototype strain as a model. The cells were infected with SARS‐CoV‐2 for 1h. After washing away the virus, different heparin or HS mimetics (Table S1, Supporting Information) were added to the infected cells separately and incubated for 48 h, as illustrated in **Figure** **1**A. The expression of viral S protein in the cells was utilized to assess SARS‐CoV‐2 infection. HMSA‐06‐20 exhibited a more robust inhibition against SARS‐CoV‐2 than other compounds tested in parallel (Figure 1B). To further confirm that HMSA‐06 can suppress SARS‐CoV‐2 infection post‐virus entry, we first infected Vero E6 cells with SARS‐CoV‐2 for 1 h, followed by additions of different doses of HMSA‐06 under non‐toxic concentrations (Figure S1, Supporting Information). HMSA‐06, or poly(SS‐*co*‐AA), is a random copolymer of sodium styrene sulfonate and acrylic acid prepared by Reversible addition−fragmentation chain‐transfer (RAFT) polymerization.^[^ ^24^ ^]^ In common with HS, HMSA‐06 contains both sulfonate and carboxylate groups (Figure 1C). To examine whether the number of repeating units impacts the antiviral effects, we included two versions of HMSA‐06 polymers, HMSA‐06‐5 and HMSA‐06‐20, with an average molecular weight (*M*_n_) of 15.2 and 29.9 kDa, respectively, as determined by GPC.^[^ ^24^ ^]^ Both HMSA‐06‐5 and HMSA‐06‐20 robustly impeded the expression of S protein (Figure 1D,E) and viral RNA (E gene) (Figure 1F) of SARS‐CoV‐2 in a dose‐dependent manner. Furthermore, in the more physiologically relevant Calu3 cell line, epithelial cells from lung tissue, HMSA‐06‐5 and HMSA‐06‐20 showed a comparable dose‐dependent inhibition of SARS‐CoV‐2 infection when the compounds were added to the cells post‐viral infection (Figure 1G).

To evaluate the potential of HMSA‐06 in inhibiting SARS‐CoV‐2 cell entry, we performed a standard plaque assay. HMSA‐06‐20 showed ≈50% inhibitory effect on SARS‐CoV‐2 cell entry at a concentration as low as 0.25 µm (Figure 1H). HMSA‐06‐5, however, did not exhibit an apparent effect on SARS‐CoV‐2 cell entry, suggesting that the molecular weight of HMSA‐06 is associated with its function in blocking SARS‐CoV‐2 entry. In comparison, another HS mimetic polymer, HMS‐01 or poly(SS),^[^ ^24^ ^]^ with a similar structure to HMSA‐06, was essentially unable to inhibit SARS‐CoV‐2 entry except for HMS‐01‐20 at the concentration of 1 µm (Figure 1I). It is also worth noting that more net negatively charged heparin (having a similar structure to HS) failed to inhibit the cell entry of the SARS‐CoV‐2 prototype (Figure S2A, Supporting Information). Thus, the results point to a specific effect of HMSA‐06 in the restriction of the SARS‐CoV‐2 prototype cell entry and replication.

### 2.2 HMSA‐06 Restricts the SARS‐CoV‐2 Omicron Infection

**Figure 2:**
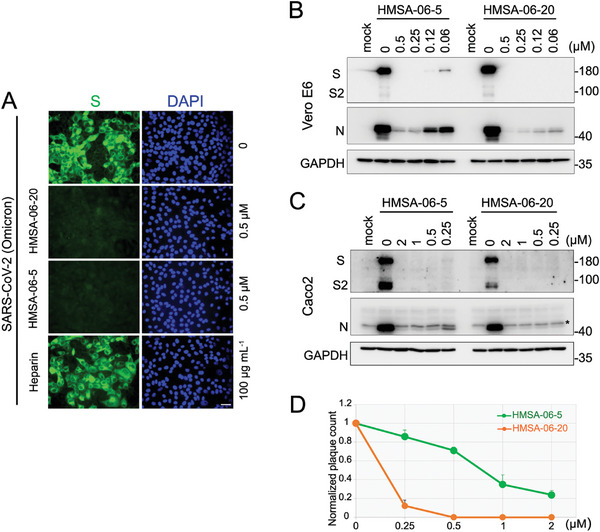
HMSA‐06 restricts the SARS‐CoV‐2 Omicron infection. A) Vero E6 cells were infected by SARS‐CoV‐2 Omicron (MOI=0.5), and HMSA‐06‐20 (0.5µm) or heparin (100µg mL^−1^) were added to the medium at 1h after infection. Cells were fixed at 48 h post‐infection, and the expression of the SARS‐CoV‐2 Omicron S protein in the presence or absence of HMSA‐06 or heparin was examined by Immunofluorescence assay. The representative images from two independent experiments were shown. Scale bar, 30µm. B) Vero E6 cells or C) Caco2 cells were treated with different concentrations of HMSA‐06 as indicated after infection with SARS‐CoV‐2 Omicron (MOI=0.01). The expression of the SARS‐CoV‐2 Omicron S protein and nucleocapsid protein (N) were evaluated by western blot. The representative western blots of two independent experiments were presented. The non‐specific bands detected by the anti‐SARS‐CoV‐2 N protein antibody in the Caco2 cells were indicated by an asterisk. D) The number of plaques formed by the SARS‐CoV‐2 Omicron infection with or without HMSA‐06 incubation at different concentrations. The data were normalized by the plaque number in the absence of HMSA‐06 from two independent experiments.

During our study, the Omicron variant outcompeted the previous SARS‐CoV‐2 variants and emerged as the dominant strain due to its higher transmissibility and immune evasion capacity. The unique nature of Omicron attracted us to investigate whether HMSA‐06 could also mitigate Omicron infection. Using the same experimental settings of post‐infection treatment, HMSA‐06 robustly reduced the expression of the S protein by immunofluorescence staining (**Figure** **2**A) and the expression of S and N proteins by Western blot (Figure 2B) in Vero E6 cells, even at a concentration as low as 0 .25µm. Again, heparin failed to show any apparent effects against Omicron infection (Figure 2A), confirming our assumption that HMSA‐06 owns a unique feature different from heparin and independent of total negative charge. To investigate if the anti‐Omicron effect of HMSA‐06 is cell specific, we also utilized Caco2 cells (epithelial cells isolated from human colon tissue) as the model. Similarly, HMSA‐06 exhibited a significant inhibitory function against the SARS‐CoV‐2 Omicron infection (Figure 2C). Additionally, HMSA‐06, especially HMSA‐06‐20, significantly blocked the entry of the SARS‐CoV‐2 Omicron (Figure 2D).

Taken together, our results demonstrated that HMSA‐06 could significantly impede SARS‐CoV‐2 (both the prototype and Omicron strains) infection by blocking viral entry and disturbing viral replication in different cell models.

### 2.3 HMSA‐06 Exhibits Higher Anti‐SARS‐CoV‐2 Activities than PG545 and Roneparstat

**Figure 3:**
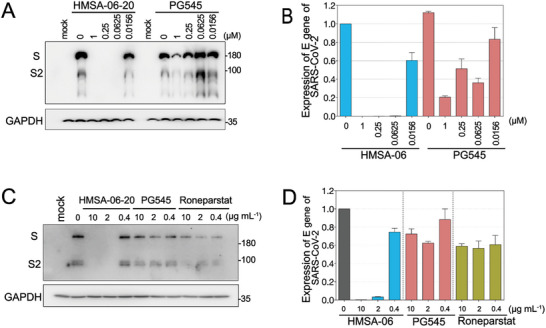
HMSA‐06 exhibits higher anti‐SARS‐CoV‐2 activities than PG545 and Roneparstat. A) The experiments were set up in a similar way as in Figure 2B. The representative western blots illustrate the expression of the SARS‐CoV‐2 Omicron S protein in the presence or absence of different concentrations of HMSA‐06‐20 and PG545 in Vero E6 cells. B) The mRNA expressions of the SARS‐CoV‐2 E gene from the same samples in (A) were assessed by q‐PCR and data were shown as mean ± SD from two independent experiments. C) The representative western blots show the expression of the SARS‐CoV‐2 Omicron with or without treatments of different concentrations of HMSA‐06‐20, PG545, and Roneparstat in Caco2 cells. D) The mRNA expressions of the SARS‐CoV‐2 E gene from the same samples in (C) were assessed by q‐PCR and data were shown as mean ± SD from two independent experiments.

While investigating the mechanisms by which the HMSA‐06 inhibits SARS‐CoV‐2, PG545 (pixatimod) and Roneparstat (two HS mimetics that were originally developed for cancer) were reported to have inhibitory effects against SARS‐CoV‐2 infection.^[^ ^23^, ^25^ ^]^ We compared the inhibitory activities of HMSA‐06‐20 with PG545 and Roneparstat for inhibition of SARS‐CoV‐2 Omicron in the Vero E6 and Caco2 cell models. As shown in **Figure** **3**, HMSA‐06‐20 almost completely inhibited the SARS‐CoV‐2 Omicron replication at as low as 0.0625 µm (Figure 3A,B) in Vero E6 cells and 2 µg mL^−1^(Figure 3C,D) in Caco2 cells, while PG545 and Roneparstat only partially impeded SARS‐CoV‐2 Omicron replication at the same concentrations. The results indicate that HMSA‐06‐20 is superior to PG545 and Roneparstat with regard to anti‐SARS‐CoV‐2 Omicron infection.

### 2.4 HMSA‐06 Boosts Autophagy

**Figure 4:**
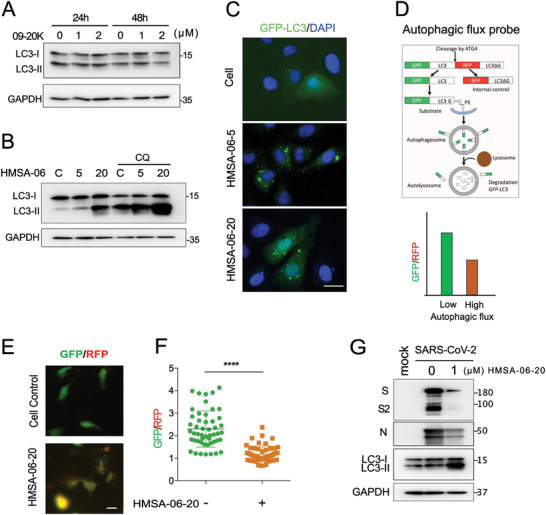
HMSA‐06 boosts the autophagy. A) The 70%–80% confluent Vero E6 cells were incubated with 09–20K (1 and 2 µm) for 24 and 48 h, the expression of LC3 was assessed by western blot. The representative western blots of three independent experiments were shown. B) The 70%–80% confluent Vero E6 cells were incubated with HMSA‐06‐05 or HMSA‐06‐20 (2 µm) for 48 h. Or CQ (20 µm) was added to the cells at 40 h after HMSA‐06 treatment. The representative western blots of three independent experiments were shown. GAPDH was utilized as a loading control. C) GFP‐LC3 was transfected into Vero E6 cells. After 24 h, the cells were incubated with HMSA‐06‐20 (2 µm) for 24 h and CQ (20 µm) was added to the cells at 16 h after HMSA‐06‐20 treatment. The GFP‐LC3 puncta in the cells in the absence or presence of HMSA‐06 were observed under the microscope. The representative pictures were shown. Scale bar, 20 µm. D) The working mechanism of the autophagic flux probe adapted from the previous study^[^ ^26^ ^]^ is shown. A lower GFP/RFP ratio indicates higher autophagic flux. E) The probe pMRX‐IP‐GFP‐LC3‐RFP‐LC3ΔG was transfected into Vero E6 cells and treated with or without HMSA‐06‐20 (2 µm) for 24h. Cells were fixed and the GFP and RFP fluorescence were observed under a microscope. The representative images that merged by GFP and RFP fluorescent channels were shown. F) Quantification of GFP/RFP fluorescence intensity. The Mean ± SD of the ratio of GFP and RFP fluorescence intensity in 50 transfected cells recorded in each condition is shown (*n* = 50). Scale bar, 20 µm. Statistical analysis was performed using Student's t‐test. ^****^ *P* ≤ 0.0001. G) The representative western blots of two independent experiments illustrating the expression of the SARS‐CoV‐2 S protein, N protein and cellular LC3 with or without treatment of HMSA‐06‐20 were shown. GAPDH was used as a control.

The antiviral function of HS or HS mimetics is thought to be mainly attributed to their roles in blocking viral entry, as HS is shown to be a co‐receptor of many viruses. As we found that HMSA‐06 was able to reduce SARS‐CoV‐2 replication even after the cells were infected by the virus regardless of the molecular weight (Figure 1), we hypothesized that HMSA‐06 may remodel some cellular processes that are important for SARS‐CoV‐2 replication. Autophagy is closely involved in different stages of viral replication and accumulating evidence shows that induction of autophagy impaired SARS‐CoV‐2 replication.^[^ ^22^, ^27^ ^]^ To investigate whether HMSA‐06 has any effects on autophagy, we treated cells using different doses of HMSA‐06‐05 or HMSA‐06‐20 and monitored the expression of LC3II, a standard marker for autophagosomes, at different time points post‐treatment. Both HMSA‐06‐05 and HMSA‐06‐20 upregulated the expression of autophagy marker LC3II and HMSA‐06‐20 showed a more potent boost (Figure S3, Supporting Information). In contrast, the HS mimetic 09–20K (also known as poly(SPA‐*co*‐AA)), which has a similar overall charge and molecular weight to HMSA‐06‐20 (Table S1, Supporting Information) and did not inhibit SARS‐CoV‐2 infection (Figure 1B), failed to alter LC3II expression (**Figure** **4**A). To further clarify whether HMSA‐06 increased the autophagy flux or blocked the autophagy, we treated the cells with HMSA‐06 in the presence or absence of the autophagy inhibitor, chloroquine (CQ). In the presence of HMSA‐06, CQ treatment failed to curb the increase of LC3II (Figure 4B). This was further proved by the observation that an enhanced number of GFP‐LC3II puncta was seen in the HMSA‐06 treated cells (Figure 4C). In addition, we used an autophagy probe, pMRX‐IP‐GFP‐LC3‐RFP‐LC3ΔG, whose working mechanism is illustrated in Figure 4D ^[^ ^26^ ^]^ to check the effects of HMSA‐06‐20 on cellular autophagic flux. The ratio of fluorescence GFP and RFP intensity was significantly decreased in the presence of HMSA‐06‐20, suggesting that HMSA‐06‐20 enhanced autophagic flux (Figure 4E,F). The upregulation of autophagy by HMSA‐06‐20 treatment is correlated with the marked decrease in SARS‐CoV‐2 S and N protein expression after infection (Figure 4G), strongly supporting the hypothesis that the effect of HMSA‐06 in attenuation of virus replication is associated with its activity in boosting autophagic flux.

### 2.5 HMSA‐06 Attenuates the Inflammatory Reaction Induced by SARS‐CoV‐2 Infection

Hyperinflammation profoundly impacts the severity of COVID‐19 and NLRP3‐mediated inflammatory reaction plays a critical role in the pathological process.^[^ ^28^ ^]^

**Figure 5:**
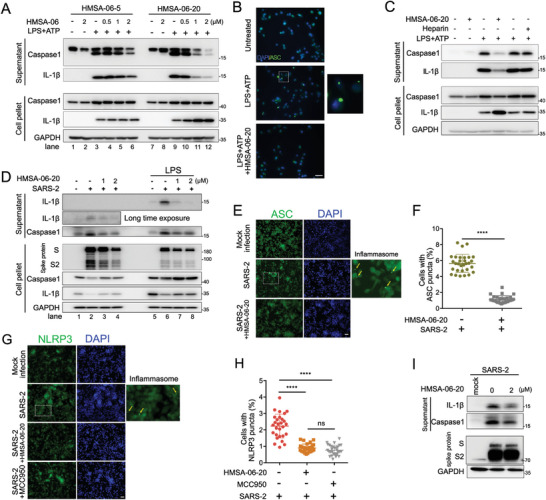
HMSA‐06 attenuates the inflammatory reaction induced by SARS‐CoV‐2 infection. A) The macrophage‐like THP‐1 cells were induced by LPS+ATP to activate the inflammation reaction in the presence or absence of various concentrations of HMSA‐06‐20 or HMSA‐06‐05. The expression of the mature caspase‐1 and IL‐1β in the supernatant as well as the pro‐caspase‐1 and pro IL‐1β in the cell pellets were assessed by western blot. The representative western blots of two independent experiments were shown. B) Macrophage‐like THP‐1 cells were induced by LPS+ATP in the absence or presence of HMSA‐06‐20. The ASC puncta were observed under the microscope and the representative pictures were shown. HMSA‐06 significantly decreased the number of ASC puncta. Scale bar, 30 µm. C) The macrophage‐like THP‐1 cells were treated with LPS+ATP to activate the inflammation reaction with or without adding HMSA‐06‐20 (2 µm) or heparin (100 µg mL^−1^). The expression of pro‐ or mature caspase‐1 and IL‐1β in cell pellets or supernatants were assessed by western blot. D) Macrophage‐like THP‐1‐ACE2 cells were infected by the SARS‐CoV‐2 prototype (MOI = 0.2) in the absence or presence of HMSA‐06‐20 (1 or 2 µm) with or without prime by LPS. The western blots show the expression of the pro or mature caspase‐1 and IL‐1β in cell pellets or supernatant as well as the expression of the SARS‐CoV‐2 S protein. The representative western blots of two independent experiments were shown. E) The representative images illustrate the ASC puncta in the SARS‐CoV‐2 infected macrophage‐like THP‐1‐ACE2 cells with or without adding the HMSA‐06‐20. Scale bar, 30 µm. F) Quantification of the cells with ASC puncta. The Mean ± SD of the percentage of the cells with ASC puncta from 30 images randomly taken from each condition is shown (*n* = 30). Statistical analysis was performed using Student's t‐test. ^****^ *p* ≤ 0.0001. G) The representative images show the NLRP3 puncta in the SARS‐2 (SARS‐CoV‐2) infected macrophage‐like THP‐1‐ACE2 cells in the presence or absence of HMSA‐06‐20 (2 µm) or MCC950 (10 µm). Scale bar, 30 µm. H) The Mean ± SD of the percentage of the cells with NLRP3 puncta from 30 images randomly taken from each condition in (E) is shown (*n* = 30). Statistical analysis was performed using Student's t‐test. ^****^ *p* ≤ 0.0001. ns, not significant. I) The macrophage‐like THP‐1‐ACE2 cells were infected using a high titer of SARS‐CoV‐2 (MOI = 5). The expression of active caspase‐1 and mature IL‐1β in the supernatant and the expression of SARS‐CoV‐2 S protein in cell pellets in the presence or absence of HMSA‐06‐20 (2 µm) were examined by western blot.

Since heparin has been reported to have anti‐inflammatory activities,^[^ ^29^ ^]^ we wanted to find out whether HMSA‐06 has an anti‐NLRP3 mediated inflammatory reaction. Firstly, we used the THP‐1‐derived macrophage as a cell model. Cells were primed with Lipopolysaccharide (LPS) followed by the stimulation using ATP to activate NLRP3 inflammasome, which resulted in the formation of active caspase‐1 and subsequent mature IL‐1β (**Figure** **5**A lane 3 and lane 9). Both HMSA‐06‐05 and HMSA‐06‐20 dose‐dependently decreased the active caspase‐1 and mature IL‐1β that released into the supernatant medium, though HMSA‐06‐20 showed more potent inhibitory effect (Figure 5A lanes 3–6 and lanes 9–12). This finding is confirmed by the observation that the number of ASC puncta in the cells was markedly reduced in the presence of HMSA‐06‐20 (Figure 5B). Heparin possesses a similar structure to HS. To assess if heparin could also hinder the NLRP3‐mediated inflammatory reaction, we performed a similar experiment as in Figure 5A. HMSA‐06‐20 exhibited a robust inhibition of mature IL‐1β and caspase‐1, while heparin did not show any effects (Figure 5C).

Infection of THP‐1‐derived macrophage by SARS‐CoV‐2 has been shown to activate NLRP3 inflammasome.^[^ ^21^ ^]^ To further assess whether HMSA‐06 could also hamper the NLRP3‐mediated inflammatory response induced by SARS‐CoV‐2, we used a THP‐1 stably expressing ACE2 cell model (THP‐1‐ACE2). THP‐1‐ACE2 cell‐derived macrophages were infected by SARS‐CoV‐2 with or without LPS priming. As expected, SARS‐CoV‐2 infection induced the expression of active caspase‐1 and mature IL‐1β in the supernatant (Figure 5D, lane 2 and lane 6), which was effectively inhibited by HMSA‐06‐20 in a dose‐dependent manner (Figure 5D, lanes 2–4). This result was further proved by SARS‐CoV‐2′s infection under LPS priming. SARS‐CoV‐2 infection induced more mature IL‐1β after LPS priming and HMSA‐06 significantly decreased the IL‐1β in the supernatant (Figure 5D, lanes 6–8). Additionally, we observed that the number of ASC puncta induced by SARS‐CoV‐2 was remarkably decreased in the presence of HMSA‐06‐20 (Figure 5E,F). In agreement with these data, the HMSA‐06‐20 treatment also resulted in a significant reduction of NLRP3 puncta (Figure 5G,H). This effect of HMSA‐06‐20 was almost the same as MCC950 that is a selective inhibitor of the NLRP3 inflammasome (Figure 5G,H). These data suggest that HMSA‐06 could prevent the NLRP3 inflammatory reaction triggered by SARS‐CoV‐2 infection.

HMSA‐06 was able to inhibit SARS‐CoV‐2 replication in different cell lines (Figures 1 and 2). HMSA‐06‐20 showed a dose‐dependent inhibition against SARS‐CoV‐2 infection in the THP‐1 cell model (Figure 5D). To investigate whether the reduction in SARS‐CoV‐2 infection by HMSA‐06‐20 entirely resulted in the downregulation of IL‐1β, we infected THP‐1‐ACE2 cells with a high titer of SARS‐CoV‐2 in the presence or absence of HMSA‐06‐20. As presented in Figure 5I, HMSA‐06‐20 marginally affected the SARS‐CoV‐2 infection, but it markedly attenuated the IL‐1β released into the supernatant. In addition, HMSA‐06‐20 can also attenuate the IL‐1β induced by the SARS‐CoV‐2 Omicron (Figure S4, Supporting Information). In consideration of the fact that HMSA‐06‐20 was able to attenuate the LPS‐induced inflammatory reaction, collectively, the data indicate that HMSA‐06 may pose an anti‐inflammatory reaction by attenuating the activation of NLRP3 inflammasome, regardless of SARS‐CoV‐2 infection.

## 3 Discussion

The unprecedented success of mRNA vaccines in mitigating SARS‐CoV‐2 infection saved hundreds of thousands of lives during the COVID‐19 pandemic. However, due to the unique characteristics of coronaviruses, new viral variants can easily compromise vaccines’ protection. Despite the fact that the available drugs targeting the virus itself, such as Paxlovid, have shown robust clinical efficacies in reducing hospitalizations and mortality of COVID‐19 patients, we are facing challenges due to the emergence of drug‐resistant strains and a relatively narrow time window for drug administration. New drugs with multiple targets, especially for targeting host factors, will dramatically reduce the possibility of the emergence of drug‐resistant SARS‐CoV‐2 strains. In addition to blocking SARS‐CoV‐2 replication, reducing the virus‐induced hyperinflammation would provide another key protective line against the development of severe COVID‐19. In this study, our identified HMSA‐06 owns capacities of both targeting different variants of SARS‐CoV‐2 and, most interestingly, attenuating NLRP3‐mediated inflammatory reaction, which provides a good candidate for the development of new antiviral drugs against coronavirus‐related diseases and beyond.

HS is a negatively charged polysaccharide ubiquitously expressed on all cell surfaces, functioning as co‐factors of many viruses, including SARS‐CoV‐2.^[^ ^30^ ^]^ The interaction between the positively‐charged viral glycosylated protein and the negatively‐charged HS is believed to promote virus accumulation on the cell surface and the subsequent viral entry. In our study, more negatively charged heparin did not exhibit any blockage effects on the cell entry of the SARS‐CoV‐2 prototype (clinical isolate in the 2nd passage) (Figure S2A, Supporting Information). This suggests that the electric charge mediated interaction between HS and the S protein may not be the major regulator of the SARS‐CoV‐2 prototype strain cell entry. This has also been observed in a similar study, showing that early passages of clinical SARS‐CoV‐2 prototype isolates were resistant to the inhibitory effect of heparin on viral infectivity.^[^ ^31^ ^]^ Adaption of the virus in cells was accompanied by the accumulation of positive charges on the viral S protein, which increased the sensitivity to the inhibitory effects of heparin.^[^ ^31^ ^]^ During the COVID‐19 pandemic, SARS‐CoV‐2 evolved from the prototype strain to the Omicron strain with an accumulation of more positively charged amino acid mutations on the viral S protein,^[^ ^32^ ^]^ which increased the sensitivity to heparin.^[^ ^33^ ^]^ Our experiments have demonstrated that the mutations of Omicron indeed increased sensitivity toward heparin. Both HMSA‐06 (Figure 2D) and heparin (Figure S2C, Supporting Information) exhibited more robust inhibition effects on the cell entry of the SARS‐CoV‐2 Omicron than the SARS‐CoV‐2 prototype.

Most synthetic HS‐mimetic polymers are prepared from sulfonated monomers. HMSA‐06 is a random, 1:1 copolymer of sodium styrene sulfonate (SS) and acrylic acid (AA) prepared by RAFT polymerization that contains both types of negatively charged functional groups found in HS, i.e., sulfonates and carboxylates, and thus was designed to more closely mimic natural HS.^[^ ^34^ ^]^ Despite possessing similar charged groups to HS, HMSA‐06 displayed significantly higher potency in the inhibition of the SARS‐CoV‐2 prototype cell entry than heparin. This suggests that unique properties other than the charge alone, such as the non‐sugar polymer backbone with side‐chain flexibility/conformation and hydrophobicity, may contribute to the superior effects of HMSA‐06 compared to heparin. These properties of HMSA‐06 may have contributed to the repressed SARS‐CoV‐2 replication post‐viral‐infection, an effect not observed with heparin post‐treatment (Figure S2B, Supporting Information). In the case of Omicron, which carries more positive changes, heparin also exhibited an inhibitory effect (Figure S2C, Supporting Information). The discrepancy in the inhibitory effects on prototype and Omicron strains, as well as post‐infection of the viruses, suggests a different pharmacological mechanism for HMSA‐06 compared to heparin and its derivatives.

The difference between the two RAFT polymers of HMSA‐06‐20 [poly(SS‐*co*‐AA)] and HMS‐01‐20 [poly(SS)] lies in the lack of carboxylate groups in the latter (Table S1, Supporting Information), which compromised the inhibitory effects of HMS‐01‐20 on SARS‐CoV‐2 prototype entry, suggesting that carboxylate groups play a role in mediating SARS‐CoV‐2 entry. The poly(SS) of higher molecular weights (38, 100 kDa) and their respective nanoparticles have been reported to show anti‐SARS‐CoV‐2 activity by blocking the virus's cell entry.^[^ ^35^ ^]^ On the other side, HMSA‐06‐20 (*M*_n_ = 29.9 kDa), but not the lower MW HMSA‐06‐05 (*M*_n_ = 15.2 kDa), could block the cell entry of SARS‐CoV‐2, suggesting that polymer length is important for optimal activity. It is likely that further increases in MW will improve the interaction with SARS‐CoV‐2 S protein and thereby block viral entry, as seen with high MW poly(SS),^[^ ^35^ ^]^ but this remains to be determined. Interestingly, HMS‐01‐20 has also recently been shown to be a potent inhibitor of HS‐dependent mosquito‐borne viruses such as dengue virus (DENV), Yellow fever virus (YFV), Zika virus (ZIKV), and Ross River virus (RRV),^[^ ^36^ ^]^ while HMSA‐06‐20 was less potent against these viruses, indicating different structures of HS might be required to optimally interact with different enveloped‐RNA viruses.

Autophagy has an intricate relationship with SARS‐CoV‐2 infection. To facilitate its replication, SARS‐CoV‐2 may, on the one hand, need to activate autophagy to favor viral replication. The formation of double‐membrane vesicles (DMVs) from autophagy has been shown to be utilized by SARS‐CoV‐2 as RNA replication organelles.^[^ ^37^ ^]^ On the other hand, at certain stages, the activated autophagy may target viral replication.^[^ ^38^ ^]^ To cope with the detrimental effects of autophagy, SARS‐CoV‐2 has developed several strategies to block complete autophagy.^[^ ^39^ ^]^ In this sense, disrupting the viral tactic of “selectively using autophagy” by promoting complete autophagy activation could attenuate SARS‐CoV‐2 replication. Several autophagy inducer compounds, such as rapamycin, spermine, spermidine, and SMIP004, have been shown to repress SARS‐CoV‐2 replication and propagation.^[^ ^27^ ^]^ The immunomodulatory drug Niclosamide, which can induce autophagy, was also found to have the capacity to inhibit SARS‐CoV‐2 replication.^[^ ^22^ ^]^ In our study, another HS mimetic, 09–20K, which was unable to boost autophagy, also failed to hamper SARS‐CoV‐2 infection (Figures 1B and 4A). Therefore, the effect of HMSA‐06 in restraining SARS‐CoV‐2 replication may be through the reregulation of autophagy. However, we found that HMSA‐06‐20 triggered more robust autophagy than the lower MW HMSA‐06‐5 (Figure S3, Supporting Information), while the inhibitory effect on SARS‐Cov‐2 replication post‐viral infection was comparable between HMSA‐06‐20 and HMSA‐06‐05. Thus, detailed mechanisms of how HMSA‐06 restrains SARS‐CoV‐2 replication by remodeling autophagy and whether HMSA‐06 has other roles in blocking SARS‐CoV‐2 replication remain to be addressed.

**Figure 6:**
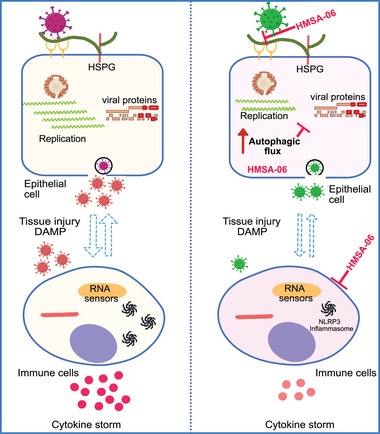
The proposed model for the anti‐SARS‐CoV‐2 infection and anti‐virus‐induced inflammation of HMSA‐06. The activation of NLRP3‐mediated inflammatory reactions has been shown in immune cells, tissues, and animal models though the mechanism of how SARS‐CoV‐2 triggers the inflammation is not fully understood.^[^ ^41^ ^]^ Based on our findings, we propose a working model describing how HMSA‐06 could act as a good candidate to treat SARS‐CoV‐2‐associated diseases: HMSA‐06 is able to inhibit SARS‐CoV‐2 cell entry (especially for the SARS‐CoV‐2 Omicron strain) and viral replication through boosting autophagic flux, which could function as the first line to prevent the progress of severe COVID‐19. In addition, HMSA‐06 significantly attenuates the activation of NLRP3 inflammasome, which could ameliorate the hyperinflammatory reaction at the late stage of SASR‐CoV‐2 infection. Damage‐associated molecular patterns (DAMP). Heparan sulfate proteoglycan (HSPG).

NLRP3‐mediated inflammation reaction drives COVID‐19 pathology, as demonstrated in different cell and animal models.^[^ ^21^, ^22^, ^28^ ^]^ NLRP3 inflammasome activation in microglia cells has also been linked to the neurological manifestation in COVID‐19 patients.^[^ ^40^ ^]^ Despite several SARS‐CoV‐2 proteins or viral replication having been implicated in inducing the NLRP3 inflammasome activation, the detailed mechanisms are not fully understood. A recent study reported that SARS‐CoV‐2 infection and replication in lung‐resident macrophages triggered the reactivation of NLRP3 inflammasome, leading to a hyperinflammatory state of the lungs and severe COVID‐19.^[^ ^16^ ^]^ Treatments with caspase‐1 and NLRP3 inhibitors significantly attenuated inflammatory cytokines in vivo and in vitro, but promoted the production of SARS‐CoV‐2 particles, which poses a risk to the benefits from the systematic inhibition of the pathway. In our macrophage model, HMSA‐06 inhibited NLRP3‐mediated inflammatory reaction as well as SARS‐CoV‐2 replication, showing dual beneficial effects. The suppression of virus replication further tones down inflammatory reactions. In this sense, HMSA‐06 possesses the capacity to balance the conflicts between the downregulation of inflammatory reaction and the upregulation of SARS‐CoV‐2 propagation, the phenomenon often seen when treating with traditional inflammatory reaction inhibitors (such as NLRP3 and caspase‐1 inhibitors). Based on the results, we propose a working model describing how HMSA‐06 could act as a suitable candidate to be developed as a comprehensive therapeutic for the treatment of severe COVID‐19 patients (**Figure** **6**).

Additionally, it is worth noting that HMSA‐06 exhibited more potent anti‐heparanase activity than PG545 which has been developed as a heparanase inhibitor.^[^ ^34^ ^]^ Heparanase has been associated with the severity of COVID‐19 in several studies^[^ ^42^ ^]^ and another heparanase inhibitor, Roneparstat, was able to decrease heparanase‐associated inflammatory reaction induced by SARS‐CoV‐2 S1 protein in vitro.^[^ ^23^ ^]^ Although the role of heparanase in the pathophysiology of COVID‐19 needs further investigation, it is clear that elevated heparanase levels damage the glycocalyx on lung epithelial cells through degradation of heparan sulfate, contributing to the pathological development in the lung.^[^ ^43^ ^]^ The higher potential of HMSA‐06 in inhibiting heparanase activity presumably will have an additive beneficial effect in managing severe pulmonary inflammation associated with SARS‐CoV‐2 infection.

Taken together, our results have demonstrated that HMSA‐06 is able to restrain SARS‐CoV‐2 infection and virus‐induced inflammation. The simple synthesis via RAFT polymerization with good MW control and low dispersity, combined with relatively low toxicity in the cells, makes HMSA‐06 a promising lead compound for developing a multiple targeting therapy for COVID‐19.

## 4 Experimental Section

### Antibodies, Chemicals and Plasmids

SARS‐CoV‐2 (COVID‐19) spike antibody [1A9] (Gene Tex, GTX632604); SARS‐CoV‐2 Nucleocapsid Antibody (R&D systems, MAB104742); GAPDH Monoclonal antibody (Proteintech, 60004‐1‐Ig); Human IL‐1 beta /IL‐1F2 Antibody (R&D systems, Catalog #: MAB201); anti‐Caspase‐1 (p20) (Adipogen, AG‐20B‐0042); NLRP3 Polyclonal antibody (Proteintech, 27458–1‐AP); ASC/TMS1 Polyclonal antibody (Proteintech, 10500‐1‐AP); LC3B Antibody (Cell signaling, 2775); Goat anti‐Mouse IgG (H+L) Secondary Antibody, HRP (Invitrogen, 31 430); Goat anti‐Rabbit IgG (H+L) Cross‐Adsorbed Secondary Antibody, HRP (Invitrogen, G21234); Goat anti‐Rabbit IgG (H+L) Cross‐Adsorbed Secondary Antibody, Alexa Fluor 594 (Invitrogen, A‐11012). Goat anti‐Mouse IgG (H+L) Cross‐Adsorbed Secondary Antibody, Alexa Fluor 488 (Invitrogen, A‐11001); Adenosine 5′‐triphosphate disodium salt hydrate (Sigma, A2383); Lipopolysaccharide (LPS) Solution (Invitrogen, 00‐4976‐93); CQ (InvivoGen, 50‐63‐5); heparin (Hepalink, Inj010702, Shenzhen); StrataClean Resin (Agilent,400714‐61); MCC950 (MedChemExpress, HY‐12815). pMRX‐IP‐GFP‐LC3‐RFP‐LC3ΔG was a gift from Noboru Mizushima (Addgene plasmid # 84 572; http://n2t.net/addgene:84572; RRID:Addgene_84572). pSELECT‐GFP‐hLC3 was purchased from InvivoGen (France, psetz‐gfplc3).

### Preparation and Characterization of the RAFT Polymers

The preparation and characterization of all RAFT polymers used in this study, including HMSA‐06, or poly(SS‐*co*‐AA) 1:1 20k, has been described previously.^[^ ^24^ ^]^ HMSA‐06 was prepared by polymerization of SS and AA in water via heating in a microwave reactor at 80 °C using BM1429 as the RAFT agent and 1,3,5‐trioxane as the internal reference for NMR analysis. The polymer was characterized by ^1^H NMR spectroscopy and GPC. Monomer conversion was determined by ^1^H NMR spectroscopy and theoretical molecular weight (18.0 kDa) was calculated using the expression:

(1) $$M_{n\;\left(\mathrm{calc}\right)}=\frac{\left({\left[M_{1}\right]}_{0}-{\left[M_{1}\right]}_{t}\right)\cdot M_{w1}+\left({\left[M_{2}\right]}_{0}-{\left[M_{2}\right]}_{t}\right)\cdot M_{w2}}{{\left[\mathrm{RAFT}\right]}_{0}}+M_{\mathrm{RAFT}}$$

M_n_ was determined by GPC to be 29.9 kDa, with *Ð* = 1.18. The unimodal peaks in the GPC chromatograms and the narrow dispersity indicate that the polymerization of SS and AA was well controlled by the RAFT agent BM1429. HMSA‐06 was confirmed to be a random copolymer of SS and AA by determination of the monomer reactivity ratios (0.1 < *r*_SS_ *r*_AA_ <1) via diverse linear least‐squares methods (Finemann‐Ross, inverted Finemann‐Ross, and Kelen‐Tudos methods). All other polymers were similarly prepared and characterized.^[^ ^24^ ^]^

### Cell lines and Virus Stains

Vero E6 (ATTC, CRL‐1586), Caco2 (ATCC, HTB‐37) and Calu3 (ATCC, HTB‐55) were cultured in Dulbecco's minimal essential medium (DMEM, Gibco) supplemented with FBS (Gibco, 10%) and penicillin (100 units mL^−1^) and streptomycin (Gibco, 100 µg mL^−1^), and grown in a 37 °C incubator with CO_2_ (5%). THP‐1‐ACE2 was a gift from Dr. Larisa Labzin, The University of Queensland. ACE2‐THP‐1 and were cultured under the conditions described previously.^[^ ^44^ ^]^ THP‐1 (ATCC, TIB‐202) was cultured in RPMI‐1640 with FBS (Gibco, 10%), penicillin (100 units mL^−1^), and streptomycin (Gibco, 100 µg mL^−1^). SARS‐CoV‐2 PM5 (SARS‐CoV‐2 prototype) and SARS‐CoV‐2 MCP43 (SARS‐CoV‐2 Omicron) were isolated directly from COVID‐19 patients using Vero E6 cells as described in.^[^ ^45^ ^]^ The passage 2 of both SARS‐CoV‐2 strains were used for the experiments in this study.

### Western Blot

The cells were lysed using RIPA buffer (Tris•HCl (pH 7.6, 25 mm), NaCl (150 mm), NP‐40 (1%), sodium deoxycholate (1%), SDS (0.1%)) supplemented with halt protease inhibitor cocktail (Thermo, 78 438). 2*Laemmli sample buffer was added into the lysate with the same amount volume and boiled at 100 °C for 10 min. The samples were fractionated in homemade SDS‐PAGE gels or acrylamide Bis‐Tris 4%–15% gradient gels (Bio‐Rad). The separated proteins on the gel were transferred onto a PVDF membrane (Millipore) and were subject to blocking by non‐fat milk (5%) in TBST buffer (TBS containing Tween‐20 (0.1%)). The primary antibody was diluted with non‐fat milk (5%) in TBST buffer and incubated for 1 h at room temperature or overnight at 4 °C on the shaker. The membrane was washed four times using TBST and followed by a 1‐h incubation with corresponding horseradish peroxidase‐conjugated secondary antibodies. After 4 times wash, the immunocomplexes were detected by Super Signal West Pico PLUS substrate (ThermoFisher) using the ChemiDoc System (Bio‐Rad).

### Quantitative PCR

RNA isolation from cell pellets was performed by RNeasy Mini Kit (Qiagen, 74 104) according to the protocol provided by the manufacturer. Around 1 µg RNA from each sample was utilized to synthesize cDNA using a High‐Capacity Reverse transcription kit (Applied Biosystem, 4 368 813) with the program: 25 °C for 10 min; 37 °C for 120 min; 85 °C for 5 min. Quantitative PCR was conducted by Power Track SYBR Green Master Mix (Applied Biosystems, A46109) using the cycling conditions: 95 °C for 30 s, followed by 45 cycles of denaturation at 95 °C for 10 s and annealing/extension at 60 °C for 60 s. Fold change was calculated as Fold Change = 2‐Δ(ΔCt) where ΔCt = Ct _target_−Ct _housekeeping_ and Δ(ΔCT) = ΔCt _treated_− ΔCt _untreated_. The primers used in this study for quantitative PCR are listed below. SARS‐CoV‐2 E gene (F‐5′TTGGTCATGATACTGCTGATTGC3′; R‐5′;CCTTCCACAAAGTCCCTATTGC3′); human GAPDH gene (F‐5′TGGGCTACACTGAGCACCAG3′; R‐5′ GGGTGTCGCTGTTGAAGTC3′).

### NLRP3 Inflammasome Activation and Caspase‐1, IL‐1β Measurement

PMA‐treated THP1 or ACE2‐THP1 cells were primed with LPS (500 ng mL^−1^) for 1.5 h. Then, cells were intensively washed four times with PBS and fresh media were added into the cells with HMSA‐06 or other compounds indicated in the figures. After 24 h, the cells were incubated with ATP (2 mm) for 1.5 h before collecting the supernatant and cell pellets. The supernatant was incubated with StrataClean resin to collect the secreted proteins as described previously.^[^ ^46^ ^]^ The caspase‐1 and IL‐1β in supernatant and cell pellets were measured by Western blot. For the inflammatory reaction in THP‐1‐ACE2 infected by SARS‐CoV‐2, THP‐1‐ACE2 cells with or without treatment by LPS were infected by SARS‐CoV‐2 for 1.5 h and followed by washing with culture media. Fresh culture media containing HMSA‐06 or other compounds were added to the cells. After 24 h, cell pellets and supernatant were harvested and analyzed as mentioned above.

### Immunofluorescence Assay

Cells were grown on round coverslips into the cell culture plate. The immunofluorescence assay was performed: cells were fixed by incubating with −20 °C methanol for 20 min. The fixed cells were washed 4 times by room temperature (RT) PBS followed by the permeabilization using 0.1% TritonX‐100 in PBS and blocking with 4% bovine serum albumin (BSA) for 1 h at RT. The primary antibodies were incubated with cells for 1 h at RT and followed by 4 times PBS wash. The proper Alexa Fluor‐conjugated secondary antibodies were added to the cells and incubated for 1 h in a dark incubation box at RT. After 4 times washing with PBS, the cells on coverslips were rinsed with distilled water and put on the microscope slide with a drop of mounting buffer containing DAPI (Vector Laboratories, Inc. Burlingame, CA, USA). The images were acquired by a fluorescence microscope (Eclipse 90i, Nikon, Tokyo, Japan). Analysis of images was performed by using the Fiji software.

### Plaque Assay

Different concentrations of HMSA‐06, HMS‐01 or heparin were incubated with SARS‐CoV‐2 (the prototype or Omicron strain) for 1 h at 37 °C. The viral mixtures were added into the monolayer Vero E6 cells in 24 well plates and incubated for 1 h at 37 °C. During incubation, the plate was gently shaken 3–4 times. Before adding 1 mL 0.8% agar media (Sigma‐Aldrich, A5431), cells were washed by culture media without FBS. After 3 to 4 days, cells were fixed and stained by 0.5% Crystal Violet Solution (Sigma‐Aldrich, V5265) for 2 h at RT. The wells with fixed cells were injected with distilled water to get rid of agar and washed several times. The number of plaques were counted.

### Statistical Analysis

Statistical tests were conducted using Graphpad Prism software (6.0). A one‐way analysis of variance (ANOVA) or a two‐tailed Student t‐test was used to determine statistical significance (^*^ *p* < 0.05, ^**^ *p* < 0.01, ^***^ *p* < 0.001, and ^****^ *p* < 0.0001).

## Conflict of Interest

The authors declare no conflict of interest.

## Author Contributions

J.Ling. performed conceptualization: lead; data curation: equal; acquired funding acquisition: equal; investigation: lead; methodology: equal; resources: equal; visualization: equal; wrote the original draft: lead; wrote – review and perform editing: equal). Å.L. acquired funding acquisition: equal; resources: lead; wrote – review and perform editing: equal. M.G. provided resources: equal; wrote – review and perform editing: equal. V.F. provided resources: equal; wrote – review and perform editing: equal). J.‐P.L. conceptualization: equal; acquired funding acquisition: lead; project administration: equal; resources: equal; wrote – review and perform editing: lead. J.Li. performed conceptualization: lead; data curation: lead; acquired funding acquisition: equal; investigation: lead; methodology: lead; resources: equal; validation: equal; visualization: lead; wrote the original draft: lead; wrote – review and perform editing: lead.

## Supporting information



Supporting Information

## Data Availability

The data that support the findings of this study are available from the corresponding author upon reasonable request.
