# Supplementary material for: A Heparan Sulfate Mimetic RAFT Copolymer Inhibits SARS‐CoV‐2 Infection and Ameliorates Viral‐Induced Inflammation
Source: Adv Sci (Weinh). 2024 Dec 16;12(6):2411737. doi: 10.1002/advs.202411737 (PMC11809384; doi:10.1002/advs.202411737)
Supplement: Supplementary file 1 — Supporting Information [file ADVS-12-2411737-s001.docx]

Supporting Information

**A heparan sulfate mimetic RAFT copolymer inhibits SARS-CoV-2 infection and ameliorates viral-induced inflammation**

*Jiaxin Ling, Åke Lundkvist, Marco Guerrini, Vito Ferro, Jin-Ping Li^*^, Jinlin Li^*^*

**Table S1. Structures of Heparan Sulfate mimetics used in this study**

| **Name** | **Structure** | **Molecular weight*^a,b^*** | **Resource** | **The concentration for screening** |
| --- | --- | --- | --- | --- |
| HMSA-06-5 |  Poly(SS-*co*-AA) | 4.7; 15.2 kDa | Nahain et al., *Biomacromolecules* **2020**, *21*, 1009−1021 |  |
| HMSA-06-20 |  Poly(SS-*co*-AA) | 18.0; 29.9 kDa |  | 1µM |
| HMS-01-5 |   Poly(SS) | 4.9; 11.5 kDa |  |  |
| HMS-01-20 |   Poly(SS) | 39.0; 25.5 kDa |  |  |
| 09-20K | 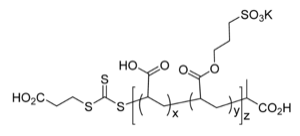  Poly(SPA-co-AA) | 18.2; 30.5 kDa |  | 1µM |
| 02-20K | 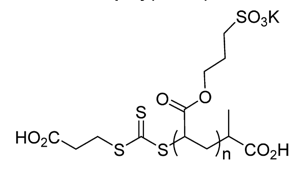  Poly(SPA) | 19.7; 23.3 kDa |  | 1µM |
| Enoxaparin | Low molecular weight heparin | 6 kDa | Commercial product | 1mM |
| A | Heparin derivative | 10 kDa | Uppsala University | 400µg mL^-1^ |
| B | Heparin derivative | 8 kDa | Uppsala University | 400µg mL^-1^ |
| C | Heparin derivative | 6 kDa | Uppsala University | 400µg mL^-1^ |
| D | Heparin fraction | 4.7 kDa | Ronzoni Institute | 400µg mL^-1^ |
| E | Heparin fraction | 4–5 kDa | Ronzoni Institute | 400µg mL^-1^ |
| F | Heparin fraction | 3.6 kDa | Ronzoni Institute | 400µg mL^-1^ |

*^a^*Theoretical MW; ^b^*M*_n_ determined by GPC.

**Supplementary methods**

**The toxicity measurement**

The effects of HMSA-06 on cell viability were assessed using PrestoBlue™ Cell Viability Reagent (Invitrogen, A13262). Vero E6 cells were grown to 70–80% in 96-well plate and treated with different concentrations of HMSA-06-05 or HMSA-06-20. After 48h, the cell viability was measured according to the protocol provided and the absorbance was recorded at 570 nm (600 nm as a reference wavelength) by EnSpire Multimode reader (PerkinElmer).

**Supplementary Figures**

**
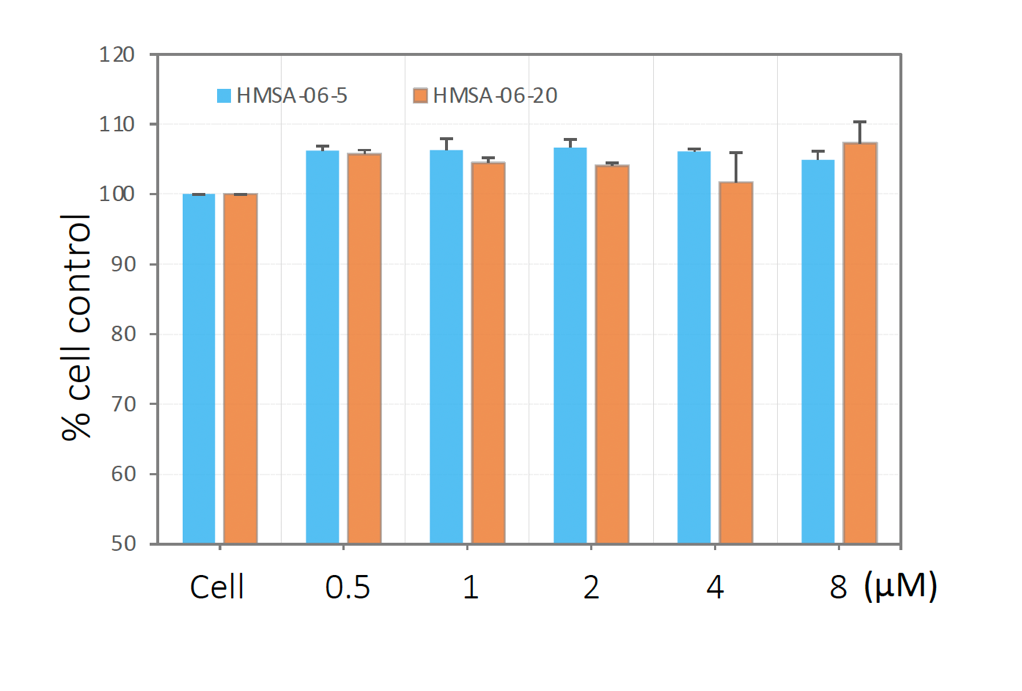
**

**Figure S1**. **The toxicity of HMSA-06.** Vero E6 cells with 70–80% confluence in 96-well plates were treated with different concentrations of HMSA-06-5 or HMSA-06-20. After 72h, cell viability was assessed by PrestoBlue™ Cell Viability Reagent according to the protocol provided by the company. The results were normalized to those samples without treatment with HMSA-06 and data were shown as Mean ± SD (n=3). HMSA-06-5 and HMSA-06-20 did not show any toxicity, even at a concentration of 8 µM.


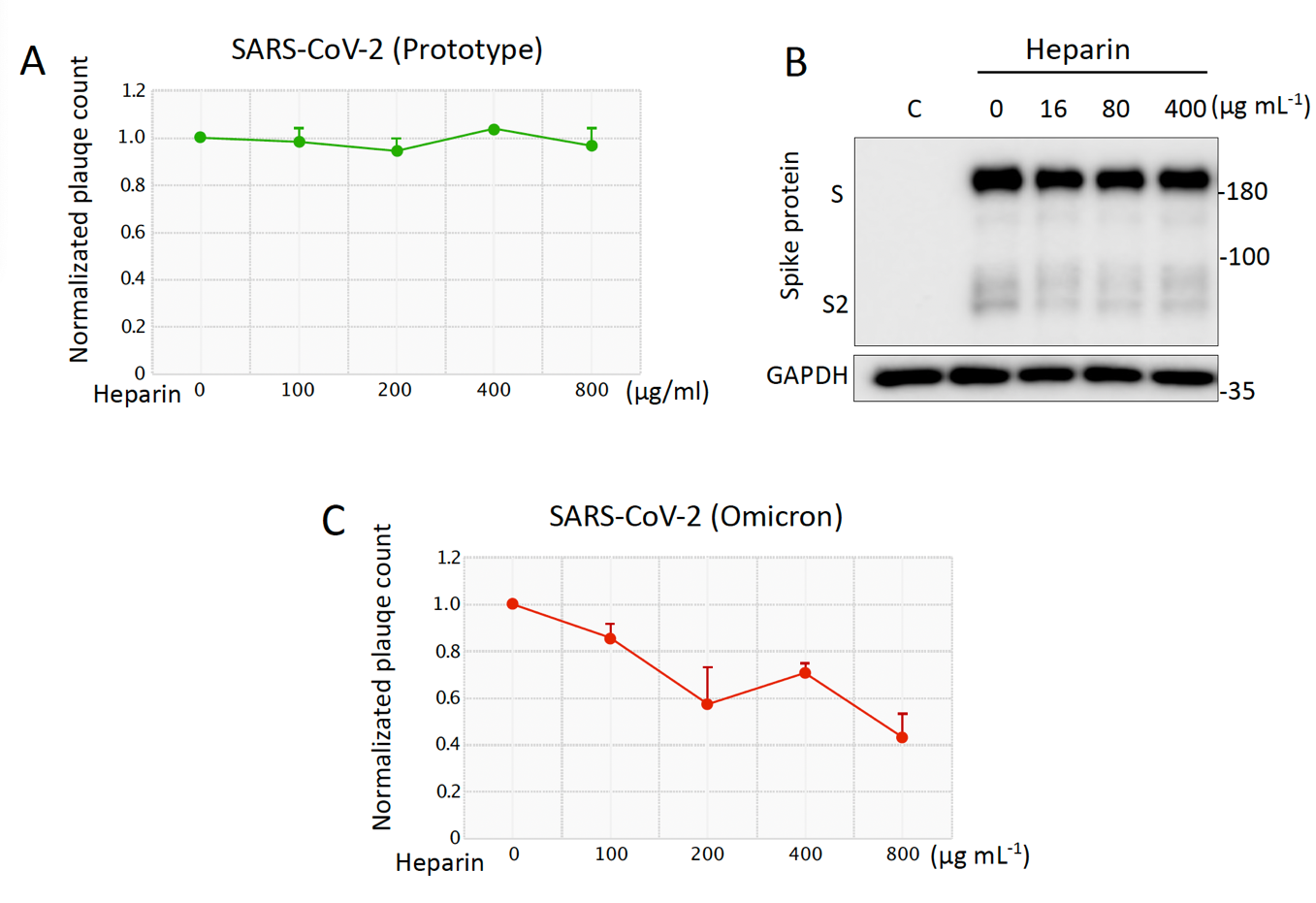


**Figure S2.** **The inhibitory effects of heparin on SARS-CoV-2 prototype and Omicron infection.** The monolayer of Vero E6 cells were infected by SARS-CoV-2 prototype (**A**) or SARS-CoV-2 Omicron (**C**) and the standard plaque assay was performed to assess the inhibitory effects of heparin on viral entry. The results were presented as relative values by normalizing with the plaque number generated in the absence of heparin. The data were shown as mean ± SD from two experiments. (**B**) Vero E6 cells were infected by SARS-CoV-2 (MOI=0.01). After 1.5h, cells were washed and the culture media with or without different concentrations of heparin were added to cells. Cells were harvested at 48h post infection and the expression of SARS-CoV-2 spike protein was evaluated by western blot using the specific antibodies indicated.


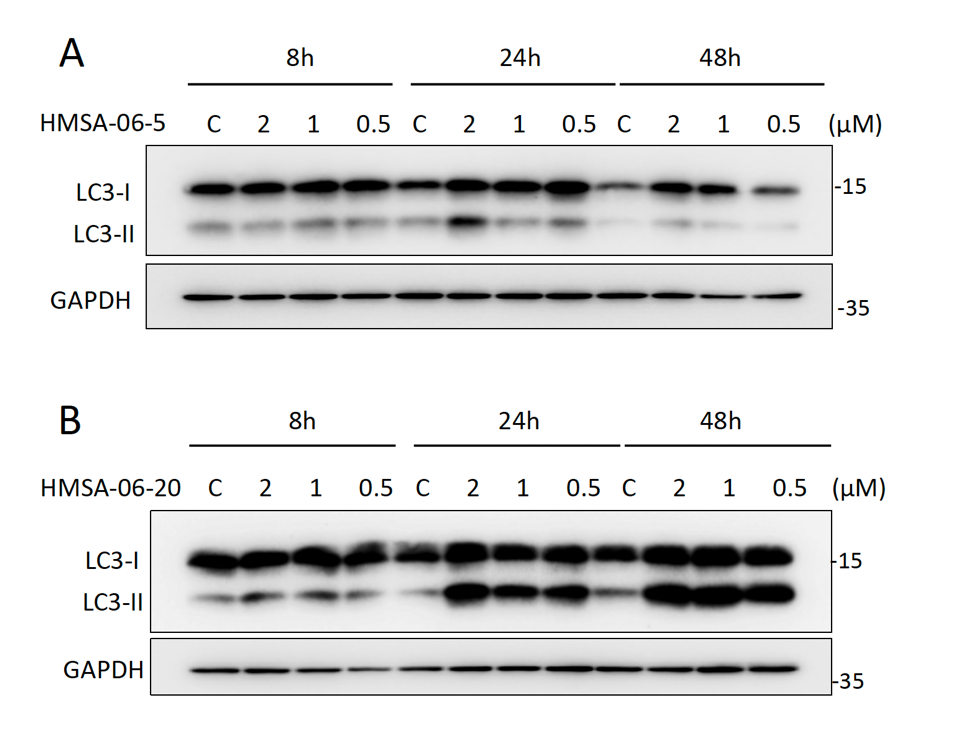


**Figure S3. HMSA-06 boosts autophagy.** Vero E6 cells were grown to 70–80% confluence. Different concentrations (0.5µM, 1µM and 2µM) of HMSA-06-5 (**A**) or HMSA-06-20 (**B**) were added into cells and cell pellets were harvested at 8h, 24h, and 48h after treatment with HMSA-06. Cells without treatment were utilized as a control (labeled as “C” in the figure). The expression of LC3I and LC3II were assessed by western blot. GAPDH was used as a loading control.


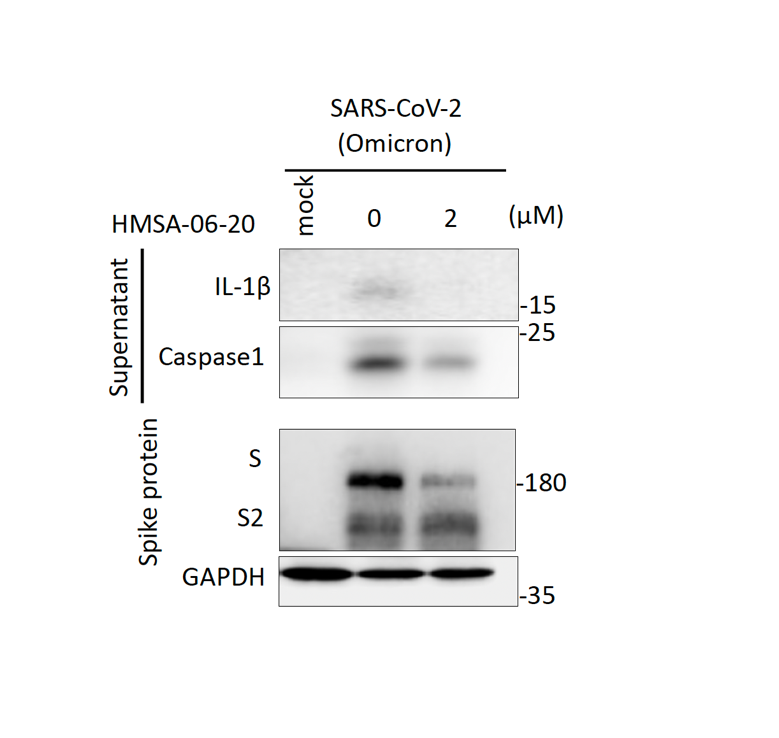


**Figure S4. HMSA-06 attenuates the active caspase** **and mature IL-1β** **induced by the SARS-CoV-2 Omicron strain.** The macrophage-like ACE2-THP-1 cells were infected by the SARS-CoV-2 Omicron. The expression of active caspase1 and mature IL-1β in the supernatant and the expression of SARS-CoV-2 S protein in cell pellets in the presence or absence of HMSA-06-20 (2 µM) were assessed by western blot.
